# Is Public Health Environmentally Sustainable?

**DOI:** 10.1007/s10728-025-00511-8

**Published:** 2025-03-04

**Authors:** Martin Marchman Andersen, Michael Z. Hauschild, Sigurd Lauridsen

**Affiliations:** 1 National Institute of Public Health, University of Southern Denmark, Odense, Denmark; 2 Department of Environmental and Resource Engineering, Technical University of Denmark, Kongens Lyngby, Denmark

**Keywords:** Sustainability, Public health, Environmental, Climate change, Planetary boundaries, Safe operating space, Environmental budgets

## Abstract

In this paper we discuss whether effective public health interventions and policies are environmentally sustainable. First, we suggest that the environmental impact from public health interventions and policies should be considered in the perspective of a human lifecycle. Second, we spell out in greater detail what we take it to mean for a public health intervention or policy to be environmentally sustainable. Third, environmental sustainability regards not only environmental impact, but also shares of our environmental “budgets”, also referred to as environmentally safe operating spaces. Such budgets represent the limits of the sustainability of a group of individuals, e.g. a population. Each individual is assigned a share of the budget for each category of environmental impact, which represents how much the individual may impact the environmental category in question without doing so unsustainably. We discuss whether individuals ought to have a larger share of these budgets as a function of their ongoing life as this would make a better case for thinking that public health interventions and policies are environmentally sustainable. But we argue that this is incompatible with maximizing health within our environmental budgets and therefore mistaken. Instead, individuals ought to be ascribed a share of these budgets for life, a share that does not increase as individuals get older. We conclude that while some public health interventions and policies might be environmentally sustainable, we cannot merely assume that public health and sustainability are win-win; indeed, we have positive reason to think that some interventions and policies are not environmentally sustainable. Finally, we elaborate on how we ought to think about and react to this conclusion.

## Introduction

In this paper we discuss whether effective public health interventions and policies are environmentally sustainable. We often have intuitive reason to think this is so. For instance, a policy of reducing the width of the street for cars and increasing it for bicycles will give people more incentive to bicycle than to drive the car, and therefore, all else equal, reduce greenhouse gas emissions *and* improve public health. Similarly, a tax on animal fat would give consumers more incentive to eat plant-based oil rather than animal fat and therefore, all else equal, promote health while reducing environmental impacts from animal farming.

If a policy is not only good for public health, but also for the environment, we may think of it as a win-win situation. It seems that more and more scholars and organizations aim to deal with environmental problems and health problems in terms of such win-win policies [1–4]. Therefore, it is interesting to consider what it takes for public health interventions and policies to be environmentally sustainable. For even though we often have intuitive reason to think that effective public health interventions and policies are environmentally sustainable, it is not necessarily the case. On that observation, considering what it takes for public health interventions and policies to be environmentally sustainable is the purpose of this paper.

First, we suggest that the environmental impact from public health interventions and policies, and indeed anything, should be considered in the perspective of a human lifecycle. We show what difference this may make to our measure of the environmental impact of interventions and policies. Second, we spell out in greater detail what we take it to mean for a public health intervention or policy to be environmentally sustainable. Third, as we shall see, environmental sustainability regards not only environmental impact, but also shares of our environmental budgets, also referred to as environmentally “safe operating spaces” (see box of concepts, 1). We discuss whether individuals ought to have a larger share of these budgets as a function of their lifetime. That is, should individuals as a function of their ongoing life, over time receive a larger share of the budget? This could be done in several ways and would make a better case for thinking that public health interventions and policies are environmentally sustainable. But we argue that this is incompatible with maximizing health within our environmental budgets and therefore mistaken, at least on that imperative. We consider different alternatives and argue that individuals ought to be ascribed a share of the budgets for life; that is, individuals ought to receive a share of the budgets which does not increase as they get older. We conclude that while some public health interventions and policies might be environmentally sustainable, we cannot merely assume that public health and sustainability are win-win; indeed, we have positive reason to think that some interventions and policies are not environmentally sustainable. Finally, we elaborate on how we ought to think about and react to this conclusion.

To avoid misunderstanding we stress the following precision of aim: We aim to conceptualize what it takes for public health interventions and policies to be environmentally sustainable and thereby in part how the environmental sustainability of public health interventions and policies should be measured. While we do think that we – as individuals and as societies – have reason to aim for environmental sustainability, we do not mean to suggest that environmental sustainability is all that is worth striving for, nor that health interventions that are not environmentally sustainable are morally impermissible. It is an open question whether we should improve public health when it conflicts with environmental sustainability, and it is beyond the scope of this paper to address that open question.


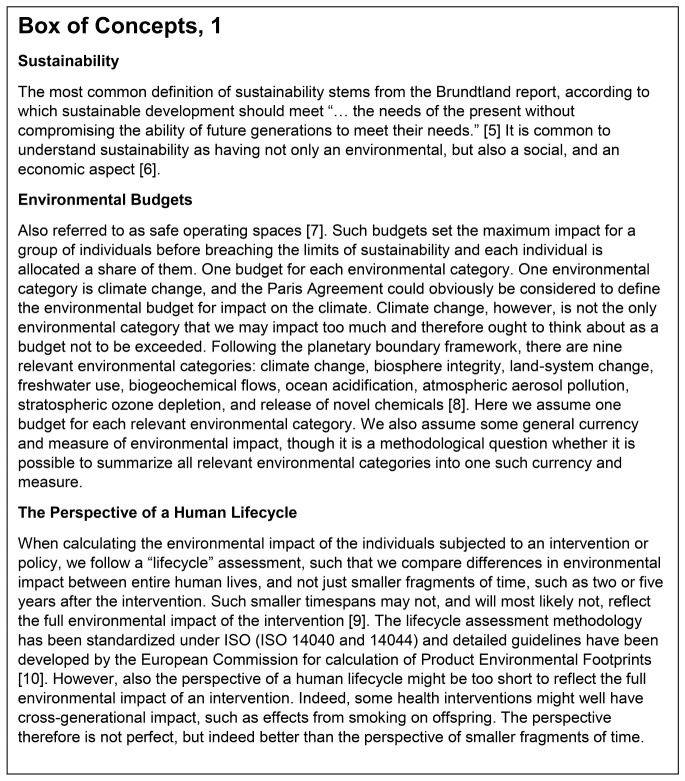


## The Importance of the Perspective of a Human Lifecycle

The “lifecycle” perspective is widely applied among environmental scientists and engineers evaluating the impact that products, services and other human activities have on the environment (9). The idea is that we need to cover impacts from products and services consumed by private (and public) consumers over their full lifecycle. From the extraction of raw materials over the production and distribution to use and final disposal or recycling of the product (including all emissions/impacts occurring in the value chain). In the same way, when considering the environmental impacts of human individuals, we should consider differences in environmental impact between entire human lives, and not just smaller time-fragments of human lives, such as two years after an intervention. This “perspective of entire human lives” makes it is less obvious that health promotion and environmental sustainability are win-win.

To exemplify, a structural public health intervention like a tax on animal fat would, all else equal, give consumers more reason to eat plant-based oil, because, all else equal, the demand will decrease when the price goes up. And since vegetable-based food in general has lower environmental impact than meat-based food, there is reason to believe that such policy would reduce environmental impact [11]. But there is also reason to hypothesize that such tax would improve the health for many [12]. When people become healthier, they tend to live longer, and when living longer they also have more time to consume and therefore, all else equal, impact the environment, e.g. by emitting more greenhouse gas.

Additionally, it is worth considering that there are large scale correlations between health and wealth [13], so when people become healthier, it is possible that they also become richer. Obviously, this is hard to verify. But if true, we should note that richer people tend to consume more and therefore, all else equal, have larger impact on the environment [14]. So, for such policy – a tax on animal fat – the question is: Do the impact reductions from a more vegetarian diet compensate the added impact from longer and more wealthy lives?

More generally, the expected consequence of a public health policy or intervention is that individuals live longer, or healthier while they are alive, or indeed both. They may also live shorter or unhealthier while they are alive, or indeed both, but no public health intervention or policy, presumably, intends to worsen public health. So, the question is whether accumulated reductions in environmental impact per time unit are larger than added impacts due to longer and healthier lives? Obviously, for each policy or intervention, this is ultimately an empirical (and methodological) question depending on specific health benefits and specific (reductions in) environmental impacts. But it seems plausible to hypothesize that some policies and interventions that immediately seem to reduce environmental impacts are in fact not net reductions when considered in the perspective of a human lifecycle. An effective non-smoking intervention or policy, for instance, would probably have this implication. For even though smoking and the production of tobacco emit greenhouse gas, the health effect of not smoking is significant. So, plausibly, an effective non-smoking intervention would increase environmental impact in the perspective of a human lifecycle. Conversely, a tax on beef would probably not have this implication and is therefore a candidate for being a win-win policy for health and environmental sustainability. The reductions in environmental impact due to the tax could be very significant and indeed relatively more so than its health effect. It is noteworthy that such implications of the lifecycle perspective would be similar when applied to interventions in other domains, such as medicine and education. However, it is beyond the scope of this paper to consider such other domains.

Now, sustainability is not only a matter of environmental impact. It is also a matter of assigning shares of our environmental budgets. To see why, we have to look into the concept of sustainability.

## What is Sustainability?

In common language, and indeed measure, sustainability is often considered as a matter of *relative* environmental impact. For instance, the environmental impact of one quantity of beef is higher than that of one quantity of pork. Therefore, in the understanding of many, the latter might seem to be the sustainable choice. However, one having smaller impact than the other is compatible with none of them being sustainable per se.

The most common definition of sustainability stems from the Brundtland report, according to which sustainable development should meet “… the needs of the present without compromising the ability of future generations to meet their needs.” [5] Note first that it obviously takes more to satisfy human needs than securing a healthy environment, and therefore it also seems common to understand sustainability as having not only an environmental, but also a social, and an economic aspect [6]. In this paper, however, we ignore these two other aspects and focus on environmental sustainability only. Henceforth, we refer to it as sustainability. Note second that sustainability regards what one generation of human beings owe to future generations of human beings. Therefore, the ultimate thing that may or may not be sustainable is generations or cohorts of human beings. As human generations or cohorts consist of and are reducible to human individuals, we should ultimately measure and think of sustainability in terms of (aggregated) sustainability of human individuals [15]. Following our precision of aim in the introduction, we should note that this does not imply that staying within the individual share of the environmental budgets is an individual rather than a common political responsibility. Here the purpose is simply to spell out how to measure and think about sustainability. It is not to actually assign rights to impact the environment. For the latter more considerations are definitely needed. Note third that sustainability is not merely relative environmental impact, but should be understood as a balance between the satisfaction of current and future needs. This means that the sustainability of an individual should be understood as a balance between its environmental impacts and its assigned share of our environmental budgets [16]. This is often referred to as *absolute sustainability* [17], that is to avoid the reading of sustainability as mere *relative impact*. We can therefore say that:

An individual is sustainable if their environmental impact is smaller than or equal to their assigned share.

The sustainability of an individual can be expressed as a ratio – impact divided by share – for each considered category of environmental impact:

If impact/share ≤ 1, then the individual is sustainable, and

if impact/share > 1, then the individual is unsustainable.

Expressing sustainability in such a ratio is useful for at least two reasons. First, it expresses not only sustainability as a dichotomy such that the individual is either sustainable or not, but also the proportionality of how sustainable or unsustainable the individual is. Second, it shows how sustainability is sensitive not only to impact, but also to the size of the shares of the environmental budgets.

Now, it seems fair to say that the environmental impact of a public health policy or intervention is the difference between the (lifecycle) environmental impact of the individuals affected by it and the (counterfactual) (lifecycle) environmental impact of the same individuals had they not been affected by the intervention. We may say that it is the difference in lifecycle impact caused by the intervention. But what then is the assigned *share* of a public health policy or intervention?

Well, we may consider interventions, policies, products, countries, institutions, companies and etc. as (un)sustainable, but we need some translation as of what this amounts to in terms of sustainability of human individuals [15]. We may say, for instance, that a product (token) is sustainable if it is consumed by a sustainable individual, that is if it is part of an individuals’ sustainable budget. Similarly, we may say that a policy or intervention is sustainable if those who are affected by it are sustainable (on average). However, first, this would depend also on all other environmental impacts of those affected, and obviously it is difficult to say much about that. Second, it is plausible to think that most individuals in Europe and North America are not sustainable, at least for now. Following for instance a recent report on Denmark’s climate footprint per capita, the sustainability ratio for average Danish consumers is 13/3, where 3/3 would be the cut off for sustainable consumption [14]. We want to be able to consider and measure the sustainability of interventions and policies in unsustainable countries. We should therefore say that:

A public health intervention or policy, P, is sustainable if those affected by it, A, are sustainable on average.

And:

If A are unsustainable on average, then P is sustainable if it decreases the average unsustainability of A.

Now P may affect the sustainability of A by affecting the impact of A. But if it increases the health of A, it may also affect the sustainability of A by affecting the assigned share of A. This is so because when A lives longer, A may have a fair claim on a larger assigned share of the environmental budgets. Let us elaborate on this point now before we then turn to our critique of it.

## Assigning Shares of the Environmental Budgets

Sustainability can be expressed as the ratio of impact divided by assigned share, and we can say that there is sustainability if the ratio is equal to or smaller than one. Thus, sustainability can be affected either by changes in impact or changes in assigned share. The relevant question now is how to assign shares of our environmental budgets, first to human individuals, and subsequently to other types of entities, such as cohorts of individuals and public health interventions and policies, products and etc.

The default position in regards to this matter of distribution is an equal per capita distribution, such that every individual gets the same share of the environmental budgets. But there might be reasons to give larger shares to individuals (in countries) who suffer from historical injustices, or to individuals who have special needs [18–19], and so on. Our point is not to settle this question here.

For now we can say that whatever else we assume, what is relevant here is mainly whether individuals should be assigned a share for life, or whether shares of the environmental budgets should be sensitive to lifetime, such that we get a larger share the older we become? This may be done in different ways, for example such that individuals are assigned a new annual share every January 1st. Philosophically speaking, this might not be a very plausible suggestion – and we do not mean to suggest that it is – because “year” is an arbitrary unit of time. We use this suggestion as an example – though an arbitrary one – of how to make shares of the environmental budgets sensitive to lifetime. We may add, in accounting the unit of a year seems to be common practice. This suggestion, rather than receiving a share for life, will affect the question of whether public health interventions and policies are sustainable. Indeed, if individuals are assigned a share every January 1st, it is more likely that public health policies and interventions are sustainable.


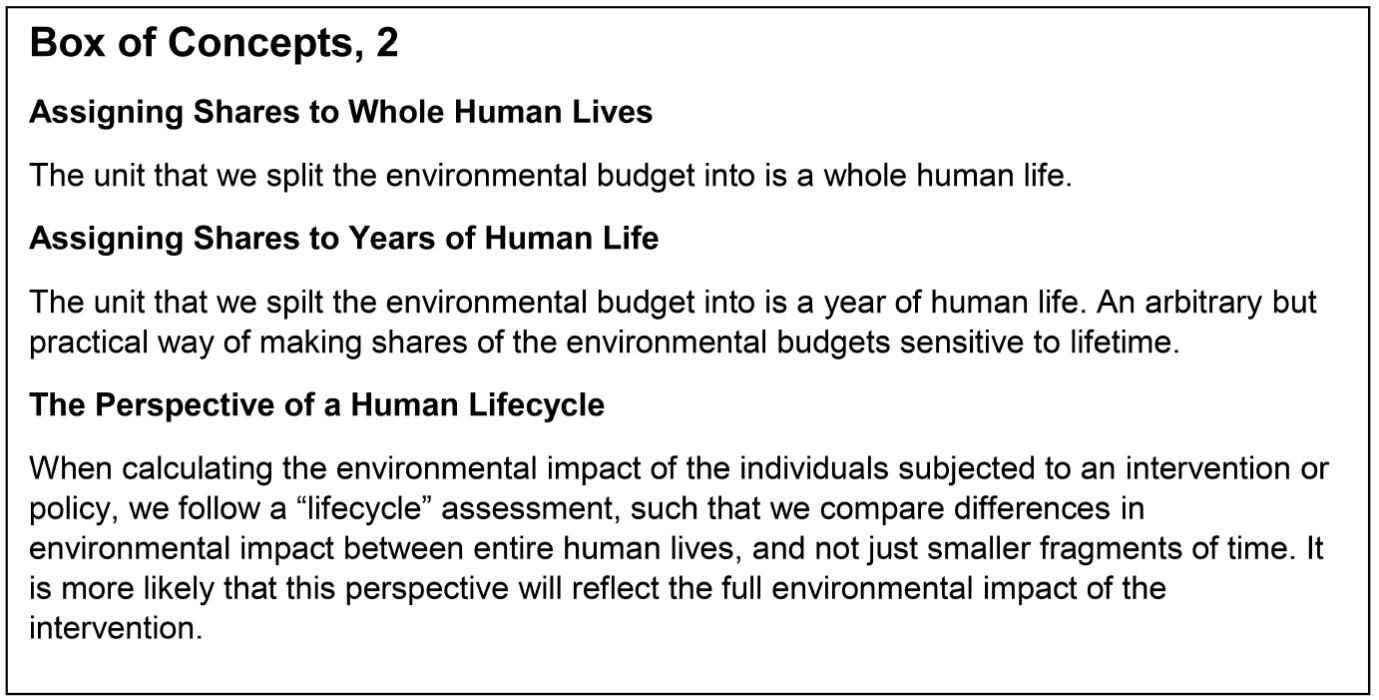


Conversely, suppose individuals are assigned a share for life. And let us again consider a tax on animal fat motivated in the promotion of public health. Would such policy be sustainable? There seems to be a higher risk that such a policy would not be sustainable, because if the impact reductions from a more vegetarian diet are smaller than the added impact from longer (and healthier) lives, there would be no additional assigned share to make up the balance. Potentially it therefore makes a difference as to whether public health policies are sustainable, if shares are assigned to whole lives rather than smaller time units.

## Making Space for New Life

What speaks in favor of letting the assigning of shares be sensitive to the duration of lifetime, as a matter of practice e.g. assigning to single years of life? Well, it might be that we are not very responsible for how long we are alive, and that with higher age (accumulated time of being alive) comes higher needs and that satisfying needs implies higher environmental impacts. This seems right. However, recalling our precision of aim in the introduction, the question here is when we should consider a life to be sustainable. This might not imply that we have obligations towards satisfying the needs of old age. It is important to remember that our responsibility towards sustainability does not exhaust morality. This means that our responsibility towards sustainability might be in conflict with other moral aims. We should obviously have some respect for human life at all ages, but that is compatible with life at some ages being more sustainable than in other ages. What might justify sensitivity to duration of lifetime?

It might be suggested that we do not, after all, owe anything to not yet existing, and therefore future, individuals. Such a view, however, would not be a plausible interpretation of sustainability, because sustainable by definition regards the satisfaction of “… the needs of the present without compromising the ability of future generations to meet their needs.” [5]. Claiming that it is not our responsibility that future generations can satisfy their needs, is thus not a take on how to understand sustainability (at least not as defined in the Brundtland report), but merely a rejection of the claim that we should care about sustainability to begin with. What then should guide us in answering the question of whether to assign shares based on whole lives or smaller time-units?

Well, a plausible take on the purpose of public health (and indeed medicine) is to get as much health as possible (ignoring discussions about negative versus positive understandings of health). We recognize from discussions of health care priority settings the imperative that we should maximize health per monetary cost [20]. Indeed, no one seems to refute this, even though not everyone agrees that this is all that matters to priority settings. If this is so, we should add that money is not the only constraint to the purpose of maximizing health. Another is environmental impact. Shouldn’t we seek to maximize health, or indeed health-related quality of life, within our sustainable budgets? If so, it seems we should set the target to be whole lives rather than smaller time-units.

This is because old lives occupy the environmental budgets, the “safe operating space” and thereby there is less for new (and younger) lives. (If new individuals come into existence and no one dies, each individual share gets smaller. If individuals die in the same frequency as new individuals come into existence, the size of shares stay constant.) As seems widely believed in discussions of health priority settings [21], younger years of life will typically contain more health-related quality of life than older years of life. This is for at least two reasons.

First, the quality of a life year should be adjusted for diseases. This is the basics in all attempts to quantify health, such as QALY and DALY. Now, diseases, especially chronic diseases, are much more prevalent in older life years than in younger ones. This is highly evident. All else equal, more diseases imply less quality of life.

Second, there is a significant difference in subjective time that matters for the quality of respectively younger and older years of life. Objectively speaking, the timespan between age 80 and age 82 is equivalent to the timespan between age 0 and age 2. Subjectively, however, this is not plausibly so. If we could count how many discrete mental events, or noticeable moments, there are packed into an objective time-slice of one year, we would probably find much more in younger persons than in older persons [21]. This indicates that the quality of life potential is higher in young life than in old life.

Accordingly, from the perspective of the *sum of quality of life* (however we define quality of life), assigning shares based on whole lives rather than smaller units of time, seems better. Conversely, defining sustainability such that individuals are assigned shares for instance for each single year they are alive, would be giving the same priority to old and less healthy years of life as to younger and more healthy years of life. Following the imperative of maximizing health, this is wrong.

Thus, the imperative to maximize health seems to favor whole lives over smaller units of time. To this imperative, however, there is another possible solution, namely to let the size of the share to the individual decrease incrementally as the individual gets older, incrementally, following the presumed decreasing development of quality of life. This solution too would imply that more public health policies would be unsustainable than if assigning the same “right to impact” to smaller units of time such as years: Generally, if the impact reductions from the intervention are smaller than the added impact from longer (and healthier) lives, there would be only very small additional assigned shares to make up the balance.

Also reasons of fairness – rather than reasons of *quality of life*-effectiveness – seem to speak against assigning to smaller units of time such as years. Looking for inspiration again in discussions over health care priority settings, we find the highly influential “fair innings” argument that all individuals should receive sufficient healthcare to provide them with the opportunity to live in good health for a normal span of years, e.g. life expectancy [22]. This implies that when resources are scarce, priority should be given to those who have not yet enjoyed good health for a normal span of years. Applying this rationale to the matter of allocating shares of our sustainable budget, it seems that allocating to whole lives rather than smaller units of time is better. This is because allocating to whole lives is to give priority to the years of life within the normal span of years, whereas allocating to smaller units of time like years implies that we are giving the same priority to life in all ages.

More generally, several scholars have shown that standard interpretations of all common distributive principles imply that higher priority should be given to younger life than to older, all else equal [21, 23]. Thus, not only reasons of health effectiveness, but also reasons of fairness seem to imply that we should not allocate equally large shares to smaller units of time such as years. Fairness, however, might not necessarily imply allocation to whole lives but might also imply that shares to old years of life should at least be discounted. It could be that the size of the share after a certain age, say life expectancy, gets significantly smaller. We conclude that in light of both reasons of health effectiveness and fairness, allocation to whole lives is a plausible suggestion, though it is not the only plausible suggestion. Allocation of equally large shares to smaller units of time like years seems mistaken.

## Conclusion

In order to preserve the satisfaction of the needs of future generations, we must incorporate the matter of sustainability in our evaluations of human activities. Public health interventions and policies are no exception. But it is not obvious how the sustainability of such activities should be considered and evaluated. In this paper we have given suggestions to some aspects of how to evaluate the environmental sustainability of public health. We repeat that we do not suggest that environmental sustainability is all there is to sustainability. There are also social and economic aspects of sustainability that may have different implications. We also repeat that we do not mean to suggest that an intervention is morally impermissible just because it is environmentally unsustainable.

Nonetheless, we have first suggested that environmental impacts should be evaluated in the perspective of a human lifecycle. This seems obvious because a human lifecycle seems better to reflect the full environmental impact of the intervention [9]. Second, we have argued that shares of the environmental budgets ought to be assigned to whole individual lives, not to smaller units of time, such as years. This is compatible with different takes on how otherwise to distribute shares of the environmental budgets.

Our suggestion as for how to evaluate sustainability is important to remember when an increasing number of scholars and organizations find common roots in environmental problems and health problems and aim to deal with them in terms of win-win policies [1–4]. It is important to remember in attempts to combine health and environmental aspects in guidance for consumers and in guidance for health politicians and decision-makers. It is important to remember more generally when contemplating about medicine, public health, and our health systems.

We suggest that sustainability is obviously relevant for discussions about priority in health. We have shown that we cannot merely assume that public health and sustainability are win-win. While some interventions and policies might be sustainable, we also have positive reason to believe that some might not be, such as for instance interventions that effectively encourage people to quit smoking.

It is worth saying that the reason as for why some public health interventions and policies are not sustainable, is that our sustainable budgets are already spent on other “consumption”. Most of us in the Western world are already unsustainable and some public health interventions and policies would make us even more unsustainable. But it might be that we, nonetheless, would give priority to some of such interventions and policies in a sustainable society leaving less budget for other “consumption”, such as eating meat, travelling by car and airplane, many web-activities, and so on. In the defense of public health, it might be suggested that such other consumption is the reason as for why some public health interventions are not sustainable. They occupy our sustainable budget. They block it for activities that arguably matter more. So, our point is not to speak against interventions that effectively make us live healthier and longer. It is rather to remind us all that when we do live longer and healthier, we ought to either reduce consumption or increase the environmental effectivity of our consumption, or indeed both. If not, we further overdraft our already environmentally unsustainable budgets.
